# Impact of Health and Health-Related Domains on Professionals’ Perceptions of Care Complexity, Their Preferences for Integrated Care Planning and Interprofessional Collaboration

**DOI:** 10.5334/ijic.8997

**Published:** 2025-07-09

**Authors:** Lisa-Maria van Klaveren, Vincent G. M. Geukers, Rien de Vos

**Affiliations:** 1 Institute for Education and Training, Amsterdam UMC, University of Amsterdam, The Netherlands; 2 Amsterdam Public Health Research Institute, Quality of Care, Amsterdam, The Netherlands; 3 Department of Pediatric Intensive Care, Emma Children’s Hospital, Amsterdam UMC, The Netherlands

**Keywords:** care complexity, integrated care, interprofessional collaboration, ICF, conjoint analysis

## Abstract

**Introduction:**

Increasing healthcare complexity necessitates the integration of perspectives from professionals with diverse expertise, patients, and families for optimal care. However, there is no consensus on ‘care complexity’, and expectations for integrated care planning vary. This study examines how different health domains influence professionals’ perceptions and preferences.

**Methods:**

Ninety-eight medical doctors and nursing professionals assessed care complexity, integrated care planning needs, and interprofessional collaboration using thirteen paper cases based on five domains from the International Classification for Functioning (ICF). Conjoint analysis explored perceptions, preferences, and variations by occupation and work experience.

**Results:**

Higher care complexity and need for integrated care planning were linked to impairments of body functions, complex personal factors in terms of chronic health condition and medical needs, and external factors. Allied health, social, and external professionals were more frequently included in multidisciplinary team meetings based on domain-specific complexities. Medical doctors showed a greater tendency than nursing professionals to involve family in integrated care planning.

**Discussion:**

The study identifies key drivers of care complexity and integrated care planning, revealing occupation- and experience-based differences. Addressing these differences is crucial for improving interprofessional collaboration.

**Conclusion:**

This research provides a multidimensional view of care complexity, highlighting the factors that shape professionals’ preferences for integrated care planning.

## Introduction

### Care complexity

Healthcare complexity is rapidly emerging across medical, situational, and organizational scales [1,2], posing significant challenges to care organization, resource allocation, and the consistent delivery of high-quality care – often framed by the three R’s: right care, right place, and right time [3,4]. Increasing medical complexity manifests as growing multimorbidity and chronicity, often coupled with the need for complex interventions and dependence on advanced technologies [5,6,7]. Beyond these medical aspects, the lived experience of complex health conditions is embedded in the persons’ personal, physical, and social environments including individual motivations, access to healthcare facilities, and support networks of family and friends [8]. The dynamic interplay between these interdependent medical, personal and environmental factors further contribute to situational complexities [9].

As a result, the delivery of care has evolved into a collaborative endeavor that surpasses individual expertise in isolated health or health-related domains. Effective care demands close cooperation among professionals across different disciplines, occupations and settings, along with active participation from patients and their families [10]. Despite this necessary shift, organizational factors – such as growing specialization and fragmentation, resource shortages (e.g., workforce limitations, inadequate training, and budgetary constraints), and entrenched hierarchical structures – continue to obstruct collective adaptation to situational complexities, further complicating the provision of optimal care [9,11]. These ongoing challenges can lead to delays in care, contradictory advice, ineffective treatments, and unsafe transitions between care settings. Ultimately, these issues undermine the patient and family experience, and adversely impact professionals’ job satisfaction, contributing to burnout and attrition [12,13].

### Integrated care planning

Integrated care has been proposed as an inter-sectorial system approach to address care fragmentation, improve care experiences, and prevent adverse effects [14,15]. At the core of integrated care are three key elements: person-focused, horizontal, and vertical integration [16]. First, person-focused integration emphasizes interprofessional collaboration between professionals, patients, and their families, promoting shared decision-making and supporting self-management [17]. Second, horizontal integration involves the formation of interprofessional care teams and networks comprising medical doctors, nursing professionals, allied health (e.g., physiotherapists), and social professionals (e.g., social workers) [17,18]. Third, vertical integration connects services across primary, community, hospital, and tertiary care settings, ensuring seamless transitions and optimal care pathways for individuals with specific conditions [17]. The appropriate extent of interprofessional collaboration – whether person-focused, horizontal, and/or vertical integration – depends on the nature of care complexity [19].

To effectively integrate perspectives, interprofessional teams must establish a shared narrative around the complexities of care and collaboratively develop strategies for integrated care planning and delivery [20]. Glader et al. [21] suggest using the International Classification of Functioning, Disability, and Health (ICF) [22] as a framework to facilitate the integration process. The ICF goes beyond the traditional biomedical model, encompassing health and health-related domains at multiple levels: the psycho-somatic (body functions and structures), the body-person (activities, personal factors), and the person-environment (participation, external factors) [23]. In clinical practice, multidisciplinary team meetings (MDTMs) are a widely used collaborative strategy to bridge professional, social, physical, and task-related gaps that can impede integrated care planning [24,25]. MDTMs serve as shared spaces for integrating the perspectives of both professionals and families into a cohesive narrative, enabling the co-creation and evaluation of integrated care plans [26]. To be effective, these meetings should include professionals with diverse expertise and actively involve families in the integrating process [27]. Furthermore, MDTMs must be conducted in a timely manner, tailored to the specific complexities of each situation, and include professionals from relevant settings [28].

### Challenges to integrated care planning

Despite the recognized importance of integrated care planning, a consensus on a general definition of care complexities and guidelines for collaborative practices in clinical care remains elusive [29]. Although care complexity, integrated care planning, and interprofessional collaboration are closely linked, significant differences in perceptions and preferences persist among professionals [30]. In particular, medical doctors and nursing professionals often have divergent views on teamwork, including aspects of involvement, workload, responsibilities, and contributions to care quality [31,32]. Similarly, the indications for and structure of MDTMs vary widely [25]. A lack of shared understanding regarding the multidimensional nature of care complexity and the collaboration it requires can lead to leadership ambiguities, unclear roles, and suboptimal decision-making [12]. These misunderstandings about roles and persistent communication challenges often originate as early as residency training [33], potentially leading to frustration, conflict, and poor teamwork [31,32].

To harmonize the perspectives and expectations of professionals with diverse expertise, it is crucial to develop a nuanced understanding of how care complexity is perceived and what preferences exist for integrated care planning. Gaining this insight can inform targeted strategies and interventions to ensure that right care is delivered in the right place and at the right time. Aligned with the quintuple aims, these improvements have the potential to enhance care experiences, reduce costs, increase professional satisfaction, and promote health equity through a comprehensive, system-wide approach to integrated care [34,35].

### Present study

The objective of this study is twofold: First, to enhance the multidimensional understanding of how varying levels of complexity across different health and health-related domains shape professionals’ perceptions of care complexity and their preferences for integrated care planning and interprofessional collaboration. Second, to examine the similarities and differences in these perceptions and preferences between medical doctors and nursing professionals, as well as across two levels of work experience. Based on these objectives, the study addresses the following research questions:

1. How does increasing complexity within individual health and health-related domains impact professionals’ perceptions of care complexity and their preferences for integrated care planning and interprofessional collaboration in terms of person-centered, horizontal, and vertical integration?
2. Does the impact of health and health-related domain complexity on these perceptions and preferences vary according to professionals’ occupation (medical doctors vs. nursing professionals) or years of work experience (≤5 years vs. >5 years)?

## Research Methods

### Design and setting

We performed an observational, cross-sectional study at the tertiary, academic Emma Children’s Hospital of the Amsterdam University Medical Centre (UMC) in the Netherlands. The Emma Children’s Hospital is organized in two distinct hierarchical lines of command for medical and nursing professionals. To facilitate integrated care throughout this organizational framework, a workplace management model is implemented consisting of a medical director and a head nurse. Functionally, interprofessional collaboration is promoted through daily ward rounds involving parents, medical and nursing staff and MDTMs that address complex patient needs. The latter are also open to multiple professionals within and across settings such as primary care, if appropriate.

### Respondents

Respondents were medical doctors and nursing professionals that worked in direct patient care at the Emma Children’s Hospital. We also included professionals-in-training (e.g., registered nurses in training for pediatric nurse, registered doctors in training for pediatrician).

### Procedure

We recruited respondents through email invitations. If needed, two reminder emails were sent at four- and eight-weeks intervals following the first invitation. After this period, one of the researchers (RV) contacted non-responding potential respondents by phone for a final request for participation. No incentives were offered for participation. Respondents were directed via a link to a web-based questionnaire in Qualtrics (Provo, UT). Here, thirteen systematically modeled patient case descriptions with varying complexities were presented in a random order to each participant. For each case, respondents were asked to answer three required and two optional follow-up questions. After finishing the last patient case, respondents answered a short series of demographic questions about their professional background and number of years of professional experience. Data were collected between December 2022 and June 2023. The study was pre-registered prior to accessing the data on the Open Science Framework (https://osf.io/6cr83).

### Case descriptions

To systematically model care complexity, we employed conjoint analysis, a scientific method recognized as optimal for assessing the value that professionals attribute to various features of products or situations in both economics and healthcare [36,37,38]. Given the Emma Children’s Hospital’s expertise of congenital and acquired disorders of the urogenital tract, we contextualized the case descriptions within the realm of pediatric urology. We modeled the case descriptions based on five health domains as outlined in the International Classification of Functioning, Disability, and Health for Children and Youth (ICF-CY) [22]: personal factors, external factors, body functions and anatomical structures, activities, and participation. In alignment with the ICF framework, we grouped body functions and structures together and addressed activities and participation separately. As the ICF does not specify *personal factors* [39,40], we followed the description by Heerkens et al. [41] for the operationalization of personal factors in terms of chronic health condition and medical needs. Within each domain, we selected two elements to represent the multidimensional nature of each domain and varied each across three levels of complexity (low, medium, high). A complete design would have included 243 case descriptions to capture the main and interaction effects of ICF-domains. Given the extensive number of combinations, we anticipated that this would place an unreasonable burden on the respondents. To address this, we generated an orthogonal design based on thirteen cases using the *conjoint* package in R (*seed* = 123). This orthogonal design allowed us to test main effects of ICF-domains on the outcome variables at the expense of interaction effects between domains. The selection of elements within each ICF-domain and their operationalization into concrete examples were carried out by a research team comprising a behavioral scientist (LK), a pediatrician-intensivist (VG), and a clinical epidemiologist (RV). Both the comprehensibility of the case descriptions, questions, and response options, and the validity and applicability of the preliminary case descriptions were verified with input from two pediatric nurses, a pediatrician, a pediatric urologist, and a physiotherapist. In multiple rounds of feedback, adjustments were made until full agreement was reached, and the materials were then finalized. Table 1 presents the operationalization of the ICF-domains.

**Table 1:** Modeled complexity by ICF-domain represented by two elements.


ICF-DOMAIN	ELEMENT	OPERATIONALIZATION (FROM LOW TO HIGH)

**Personal factors**	Chronic health condition	No health record Asthma Hypoventilation syndrome

	Medical needs	No medication Home medication Night-time non-invasive ventilation

**External factors**	Social security	Average income Welfare benefit Statutory debt restructuring

	Social network	Parents with good social network Single parent with unstable social network Divorced parent without social network

**Body functions**	Metabolic/urogenital functions	Urinates spontaneously White blood cells in urine, fever Hydronephrosis

	Sensory/mental functions	Headache Pain when urinating Pain in left flank, confused

**Activities**	Mobility	Mobilizes without support Mobilizes with support Does not mobilize

	Self-care	Sufficient oral intake Insufficient oral intake No oral intake

**Participation**	Leisure activities	Engages in team sport Engages in a solitary hobby No sport or hobby engagement

	Education	Attends regular school Attends special education High school absenteeism


#### Outcomes

To gain a multidimensional understanding of professionals’ perceptions of care complexity and preferences for integrated care planning and interprofessional collaboration, we distinguished between six outcomes measures.

##### Perceptions of care complexity

To examine the professionals’ perceptions of care complexity, we measured perceived complexity on a continuous scale from very simple (0) to very complex (100).

##### Preferences for integrated care planning

Preferences for integrated care planning were operationalized by two measures being the need for integrated care planning on a continuous scale from very unimportant (0) to very important (100) and the need for a multidisciplinary team meeting (MDTM) on a binary scale no (0) and yes (1).

##### Preferences for interprofessional collaboration

To obtain detailed insights into preferences for interprofessional collaboration, we distinguished between six groups that could potentially be included in the MDTM: parent (family), medical doctors, nursing, allied health, social professionals and external health professionals. In the context of integrated care, we conceptualize the presence of family as an indicator for person-focused integration, presence of medical doctors, nursing, allied health, and/or social professionals as indicators for horizontal integration, and external health professionals as indicators for vertical integration. Table 2 gives an overview of the outcome variables, their operationalizations in the questionnaire, and the respective scales.

**Table 2:** Overview of outcome variables with operationalizations and scales.


OUTCOME VARIABLE	OPERATIONALIZATION	SCALE

***Perception of care complexity***		

Perceived complexity	How do you evaluate the situation of the described patient at this moment?	Very simple (0) to Very complex (100)

***Preference for integrated care planning***		

Need for integrated care planning	How do you evaluate the importance of an integrated care plan for the described patient?	Very unimportant (0) to Very important (100)

Need for a MDTM	Do you think that a multidisciplinary team meeting should take place to create an integrated care plan for the described patient?	No (0) Yes (1)

***Preference for interprofessional collaboration***		

You indicated that a multidisciplinary team meeting is necessary. Who should be optional or definitely present?		

*Person-focused integration*		

Family	Parent	No (0) Optional (0) Definitely (1)

*Horizontal integration*		

Medical doctors	Pediatrician, pediatric urologist, another medical specialist	No (0) Optional (0) Definitely (1)

Nursing professionals	Pediatric nurse, pediatric nurse specialist, other nursing specialist	No (0) Optional (0) Definitely (1)

Allied health professionals	Dietician, physiotherapist, other allied health professional	No (0) Optional (0) Definitely (1)

Social professionals	Social worker, pedagogical specialist, psychologist	No (0) Optional (0) Definitely (1)

*Vertical integration*		

External health professionals	General practitioner, other external health professional	No (0) Optional (0) Definitely (1)


### Ethical considerations

Approval was provided by the Institutional Review Board of the Amsterdam UMC that waived the need for a full ethical review (W22_295#22.356). We informed respondents about the study during staff meetings, in newsletters and through email. Respondents gave written informed consent immediately before answering the questionnaire.

### Analysis

To describe the sociodemographic characteristics of the sample, we computed frequencies for professional background and training status, and median and interquartile range (IQR) for work experience.

#### Data pre-processing

Before analysis, we imputed missing data using multivariate imputation by chained equations with five imputation per outcome variable using subject id, case, professional occupation and work experience based on the R package *mice* [42].

#### Perceptions of care complexity and preferences for integrated care planning

To examine the individual effects of ICF-domains on the described outcome variables, we employed a Bayesian Multilevel Model for each outcome variable using the R package *brms* with three chains, each with 3000 iterations [43]. The ICF-domains were entered as predictors. Dummy coding was used for the three levels of complexity for each ICF-domain with low complexity as reference category. We calculated regression coefficients with a generalized Student’s t-distribution for professionals’ perceived complexity (continuous scale) and their need for integrated care planning (continuous scale), and a logistic regression with Bernoulli distribution for their need for a MDTM (binary scale).

#### Preferences for interprofessional collaboration

For interprofessional collaboration, we first calculated whether the parent or at least one professional within each group (medical doctors, nursing professionals, allied health professionals, social professionals, external health professionals) was selected as definitely present in the meeting. In case the parent or at least one professional was selected as definitely present, a score of 1 was given. Otherwise, it was coded with 0. We then used logistic regressions with Bernoulli distribution for the interprofessional collaboration of family, allied health professionals, medical doctors, nursing professionals, social professionals, and external health professionals (binary scales). Dummy coding was used for the two levels of interprofessional collaboration with 0 as reference category. To account for the repeated-measures design, we considered individual respondents as random intercept. To ease interpretation, we transformed intercepts to prior probabilities and regression coefficients for logistic regressions into odds ratios. Odds ratios indicate the increased likelihood of the integration of the parent or at least one professional within each group when complexity is medium or high versus low. We hypothesized that the impact of individual ICF-domains differed.

#### Differences between professional occupation and work experience

Finally, to assess potential differences in the impact of ICF-domains between professionals, we added professional occupation (medical doctor, nursing professional) or work experience (> 5 years, ≤ 5 years) as an interaction term to the regression models for the perception of complexity and preference for integrated care planning. The five-year cut-off point was chosen in line with earlier literature on care quality and decision-making autonomy, which identifies it as a meaningful threshold for distinguishing early-career professionals from more experienced colleagues [44,45]. To control for work experience or professional occupation in the regression models, we added them as a fixed effect.

For interprofessional collaboration, we added professional occupation and work experience only as fixed effects to the regression model. Nursing professional and more than five years of work experience were used as the reference category. To ease interpretation, we transformed intercepts to prior probabilities and regression coefficients to absolute risk reductions. Absolute risk reductions indicate the likelihood of the event (i.e., the integration of the parent or at least one professional within each group) for nursing professionals or more than five years of work experience compared to medical doctors or five or less years of work experience. Positive values indicate that medical doctors or professionals with five or less years of work experience are more likely to include family, allied health professionals, medical doctors, nursing professionals, social professionals, or external health professionals. We hypothesized that the impact of individual ICF-domains differed between nursing professionals and medical doctors, and based on work experience (> 5 years, ≤ 5 years). Statistical significance was set at the level of *p* < 0.05. All analyses were performed using R (4.0.3, 2020-10-10) [46].

## Results

### Respondents

In total, we invited 197 nurses (28 in training, 14%) and 174 doctors (54 in training, 31%) working at the Emma Children’s Hospital to take part in this observational study, of whom 117 consented to participate. We included 98 respondents (52 nursing professionals, 26%; 46 medical doctors, 26%) who completed the questionnaire for more than 50% of the cases in the final analyses. Six nursing professionals (12%) and nineteen medical doctors (41%) were in specialist training. The median professional work experience was 14 years (*IQR* = 10) for medical doctors and 7 years (*IQR* = 16) for nursing professionals. Based on their years of professional work experience, 26 medical doctors (58%) and 21 nursing professionals (40%) were placed in the high experience group (>5 years work experience).

### Perception of complexity

**Figure 1:**
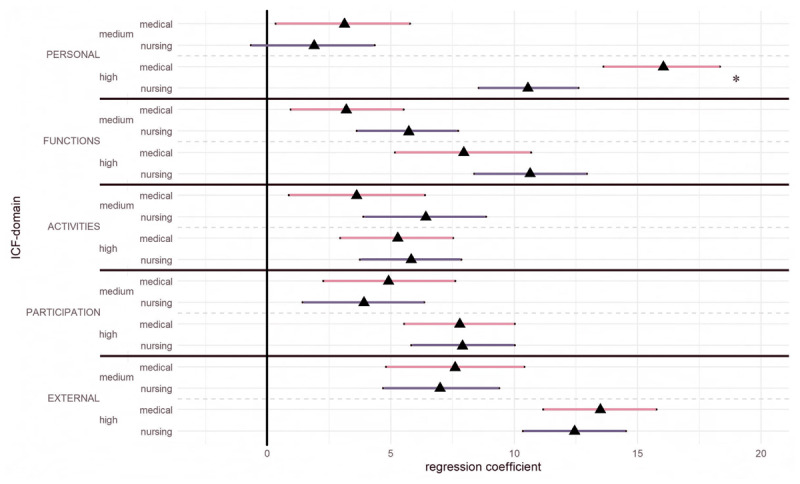
Regression coefficients and 95% confidence intervals for the impact of ICF-domains on perceived complexity for medical doctors and nursing professionals.

The analyses revealed that all five ICF-domains had a statistically significant impact on perceived care complexity. More specifically, from low to high complexity, *external factors* and *personal factors (chronic health condition, medical needs)* showed the strongest impact (*β* = 13.12, 95% CI [11.58, 14.70]; *β* = 13.08, 95% CI [11.50, 14.62]) followed by *body functions* (*β* = 9.43, 95% CI [7.65, 11.25]), *participation* (*β* = 7.86, 95% CI [6.28, 9.43]) and *activities* (*β* = 5.60, 95% CI [4.06, 7.14]). The linear regression model explained 58% of the variance in perceived complexity (*R^2^* = 57.64, 95% CI [53.45, 61.59]). Figure 1 shows the impact of individual ICF-domains on perceived complexity for medical doctors and nursing professionals.

### Preference for integrated care planning

**Figure 2:**
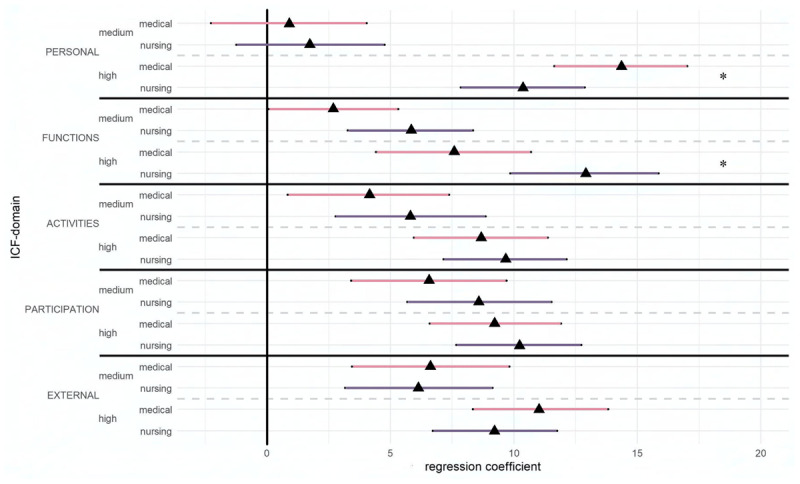
Regression coefficients and 95% confidence intervals for the impact of ICF-domains on need for integrated care planning for medical doctors and nursing professionals.

In line with perceived care complexity, all ICF-domains showed a significant impact on the need for an integrated care plan. For high compared to low complexity, *personal factors (chronic health condition, medical needs)* showed the strongest impact (*β* = 12.26, 95% CI [10.39, 14.16]). This was followed by *external factors* (*β* = 10.13, 95% CI [8.30, 11.69]) and *body functions* (*β* =10.49, 95% CI [8.37, 12.62]). The impacts of *participation* and *activities* were slightly less (*β* = 9.73, 95% CI [7.82, 11.58]; *β* = 9.15, 95% CI [7.37, 10.97]). The linear regression model explained 53% of the variance in professionals’ need for an integrated care plan (*R^2^* = 53.04, 95% CI [48.33, 57.27]). Figure 2 shows the impact of individual ICF-domains on the need for integrated care planning for medical doctors and nursing professionals.

For professionals’ need for a MDTM, the prior probability for organizing a MDTM was 51% when complexity in all ICF-domains was low (*p* = .505; 95% CI [.503, .510]). All domains showed a statistically significant impact. High complexity in *body functions* showed the highest impact (*OR* = 7.06, 95% CI [4.58, 11.16]), followed by *activities* (*OR* = 5.55, 95% CI [3.84, 8.24]). The impact of *personal factors (chronic health condition, medical needs)* (*OR* = 4.55, 95% CI [3.15, 6.64]), *external factors* (*OR* = 4.00, 95% CI [2.76, 5.78]) and *participation* (*OR* = 3.92, 95% CI [2.82, 5.54]) on the need for a MDTM was equal. The logistic regression model explained 43% of the indicated need for a MDTM (*R^2^* = 42.96, 95% CI [39.97, 45.49]).

### Preference for interprofessional collaboration

#### Person-centered integration

Family had a prior probability of 61% to be included in a MDTM when complexity in all ICF-domains was low (*p* = .613; 95% CI [.510, .987]). When *external factors* were high (compared to low), professionals were less likely to include family (*OR* = .30, 95% CI [0.09, 0.84]). The logistic regression model explained 72% of the definite family inclusion (*R^2^* = 71.71, 95% CI [68.73, 74.17]).

#### Horizontal integration

Medical doctors had a prior probability of 100% to be included in MDTMs when complexity in all ICF-domains was low (*p* = 1; 95% CI [.992, 1]). When *external factors* were complex, the odds that at least one medical doctor was included was higher (*OR* = 52.10, 95% CI [7.33, 1564.47]). The logistic regression model explained 35% of variance for medical doctor inclusion (*R^2^* = 34.27, 95% CI [23.33, 43.59]).

Similarly, nursing professional had a prior probability of 100% to be included in MDTMs (*p* = 1; 95% CI [.980, 1]). When *personal factors (chronic health condition, medical needs)* or *external factors* were complex, nursing professionals were more likely to be included in the team meeting (*OR* = 4.50, 95% CI [1.44, 15.08]; *OR* = 4.80, 95% CI [1.61, 16.59]). The logistic regression model explained 41% of the variance in nursing professional inclusion (*R^2^* = 40.79, 95% CI [30.18, 49.80]).

The prior probability for allied health professionals to be invited in MDTMs was 50% when complexity in all ICF-domains were low (*p* = .500; 95% CI [.500, .501]). When *external factors, personal factors (chronic health condition, medical needs)* or *activities* were complex (compared to low), allied health professionals were more likely to be included (*OR* = 7422, 95% CI [803.82, 23500]; *OR* = 14.90, 95% CI [3.12, 134.08]; OR = 6.51, 95% CI [1.81, 48.71]). The logistic regression model explained 63% of the variance in allied health professional inclusion (*R^2^* = 63.37, 95% CI [59.13, 66.94]).

For social professionals, the prior probability of being included was 99% when complexity in all ICF-domains were low (*p* = .986, 95% CI [.711, 1]). When *activities* or *participation* were complex, social health professionals were more likely to be included (*OR* = 9.62, 95% CI [4.39, 22.26]; *OR* = 13.98, 95% CI [6.24, 35.81]). The logistic regression model explained 46% of the variance in social professional inclusion (*R^2^* = 46.77, 95% CI [40.31, 52.09]).

#### Vertical integration

The prior probability that at least one external health professional was included in the MDTM was 50% (*p* = .500; 95% CI [.500, .502]). When *activity* was complex, external health professionals were more likely to be included (*OR* = 13.60 (95% CI [4.73, 42.18]). Conversely, when *external factors* were complex, external health professionals were less prone to be included (*OR* = .24, 95% CI [.07, .73]). The logistic regression model explained 56% of the variance in external health professional inclusion (*R^2^* = 56.28, 95% CI [50.40, 61.17]).

### Differences between professional occupation and work experience

Adding professional occupation and work experience to the model did not significantly contribute to the explained variance in perceived complexity or the need for integrated care planning. *Personal factors* in terms of *chronic health condition and medical needs* (low vs. high) had a greater impact on *medical doctors*’ perceived complexity (*β* = 5.22, 95% CI [2.13, 8.19]) and need for integrated care planning (*β* = 4.20, 95% CI [0.40, 7.90]) compared to nursing professionals. In contrast, the influence of body functions (low vs. high) on *medical doctors*’ need for integrated care planning was lower (*β* = –5.29, 95% CI [–9.69, –0.90]). No interaction effects were found for work experience. Consistent with their need for integrated care planning, *medical doctors* were more likely to organize an MDTM when *personal factors (chronic health condition, medical needs)* were complex (*OR* = 2.90, 95% CI [1.40, 6.15]) but less likely when body functions were complex (*OR* = 0.20, 95% CI [0.08, 0.50]) compared to *nursing professionals*. Additionally, *nursing professionals* and *less experienced professionals* were more inclined to plan an MDTM when *participation* complexity was high (*OR* = 0.45, 95% CI [0.22, 0.92]; *OR* = 1.06, 95% CI [1.15, 4.47]).

**Figure 3:**
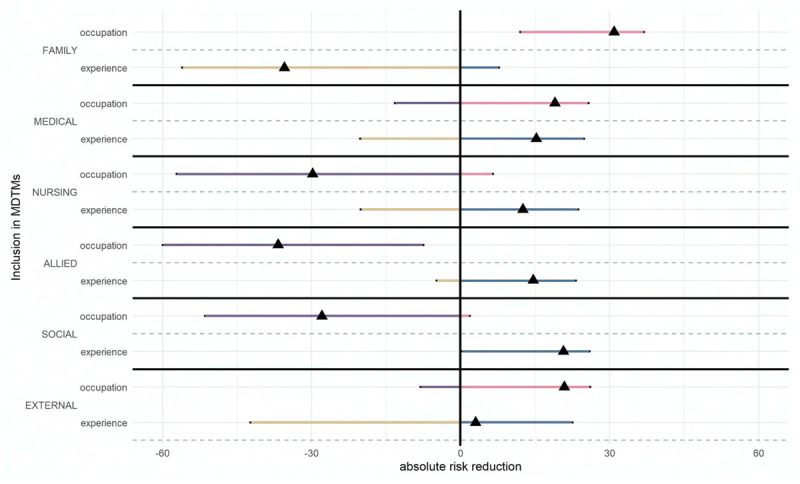
Absolute risk differences for the inclusion of family and professionals from different occupations in MDTMs between occupational group (reference: nursing professionals) and levels of work experience (reference: work experience >5 years).

*Medical doctors* were significantly more likely to include family (*OR* = 183.81, 95% CI [13.17, 7123.57]). Work experience did not influence this decision to include family. Similarly, neither profession nor work experience affected the inclusion of medical doctors, nursing professionals, or external health professionals. However, compared to *nursing professionals, medical doctors* were less inclined to include allied health professionals (*OR* = 0.20, 95% CI [0.06, 0.67]), while work experience had no impact on this choice. In contrast, respondents with *five or fewer years of experience* were more likely to include social health professionals (*OR* = 3.45, 95% CI [1.01, 12.85]). Figure 3 shows the influence of occupational group and levels of work experience on the inclusion of family and professionals from different occupations in MDTMs.

## Discussion

The objective of the study was to better understand the impact of health and health-related domains on professionals’ perceptions of care complexity, their preferences for integrated care planning and interprofessional collaboration, as well as to identify similarities and differences between professionals from different occupations (medical doctor vs. nursing professional) or with different work experience (≤5 years vs. >5 years).

### Perception of complexity and preference for integrated care planning

The results show that all ICF-domains significantly influence professionals’ perceptions of care complexity and their preferences for integrated care planning. Notably, personal factors (chronic health condition, medical needs) and external factors, along with body functions, have the greatest impact, while patients’ activities and participation are given less emphasis. This prioritization may stem from the specific focus of tertiary care settings and the scope of professionals’ expertise. First, with the shift towards distributed care networks, tertiary care is increasingly focused on managing high medical complexity, often during the acute phase of illness [47,48]. As a result, attention to activities and participation may fall outside the immediate priorities of these settings. Second, the observed differences may also reflect professional boundaries related to specific patient and environmental factors. Due to knowledge that is localized and invested within professions and settings [49], body functions and external factors might provide crucial insights for the actions of medical doctors and nursing professionals in tertiary care. In contrast, aspects such as participation in leisure activities and education might be perceived as less relevant to their professional practice [50,51].

### Preferences for interprofessional collaboration

The results also reveal that prior probabilities for person-centered, horizontal and vertical integration in interprofessional collaboration varied. Families were slightly more likely to be included in MDTMs than not, and when the complexity of external factors were low. This may reflect the general movement towards incorporating family members into integrated care planning while professionals’ decisions to adopt or reject shared decision-making seem to vary based on personal beliefs, cognitive processes, and contextual challenges [52,53]. For horizontal integration, medical doctors and nursing professionals were typically included independent from care complexity. In contrast, allied health professionals and social professionals were more frequently included in MDTMs when domain-specific complexity was high – such as personal factors and body functions for allied health professionals or activities and participation for social professionals. This aligns with the hospital’s policy of including medical doctors and nursing professionals by default, while involving allied health and social professionals as needed based on care complexity. Regarding vertical integration, external health professionals had equal chances of being involved except when complexity in activities were high. This suggests that the integration of other settings, such as primary care, is not as systematically formalized.

### Differences between professional occupation and work experience

Differences emerge between medical doctors and nursing professionals in the high-impact ICF-domains. Personal factors (chronic health condition, medical needs) have a stronger influence on the perceptions and preferences of medical doctors, while body functions are perceived as more relevant by nursing professionals. This discrepancy may reflect different knowledge and task boundaries and focal points [54]. Medical doctors might focus on longer timescales as personal factors pertained to chronic health conditions and medical needs, nursing professionals might act upon shorter timescales as body functions described the metabolic, urogenital, sensory, or mental functions at the present moment [50]. These findings suggest that professionals, drawing on their expertise, assess the contribution of health and health-related domains to care complexity and the corresponding need for integrated care planning in distinct ways.

Notably, nursing professionals were more likely than medical doctors to involve allied health professionals in MDTMs, while medical doctors showed a greater tendency to include family members. These differences might reflect nursing professionals’ views of their role as patient advocates, mediating relationships between families and other professionals. Additionally, less experienced professionals were more inclined to include social professionals during MDTMs. This may reflect a growing attention to activities and participation in integrated care planning, especially among young professionals [51]. Overall, these findings suggest that expectations regarding the composition of MDTMs vary among professionals, influenced by their specific expertise and level of experience.

Finally, the explained variance in the regression models, which ranges from 35% to 63%, indicates that while health and health-related domains, professional occupations and work experience significantly contribute to the observed differences, other critical factors remain unaccounted for. These may include individual variations beyond professional expertise, such as personal beliefs, preferences, and adherence to established standard operating procedures. For example, the routine organization of MDTMs as standard practice, regardless of care complexity, could play a role [55]. Additionally, the interdependence and dynamics between medical, situational, and organizational factors may further shape professionals’ perceptions and preferences, suggesting an influence that extends beyond individual health domains [9].

### Strengths and limitations

The findings of this study should be interpreted in light of several limitations. First, while the paper-based cases were designed to approximate professionals’ intentions in real-life scenarios, they do not fully capture real-world decision-making in complex care limiting the generalizability of the results to actual behaviors in real-world settings. However, by systematically modeling patient- and environment-related aspects within a specific clinical context, we aimed to capture the multidimensional nature of complexity, including medical, situational, and organizational scales. Despite this limitation, the use of conjoint analysis is a robust method for elucidating perceptions and preferences [38]. Additionally, the range of case complexity in this study was constrained by the selected elements within the five ICF-domains and their respective complexity levels. While the restriction to two elements provides a focused lens on multifaceted issues, challenges, and problems, it also risks oversimplification and may limit a comprehensive understanding of the broader social, economic, and political factors influencing case complexity. Although alternative frameworks exist for modeling care complexity, we chose the ICF due to its comprehensive biopsychosocial approach, which is particularly suited to addressing these multidimensional care needs.

Second, the study focused on medical doctors and nursing professionals within a tertiary care setting, which limits the generalizability of the findings to other occupations and contexts. However, insights into perceptions and preferences within a circumscribed setting with stable everyday staff composition may inform and guide further exploration of differences in the perception of care complexity and the resulting collaboration demands in other healthcare settings. Future research could explore these dynamics in different clinical environments, involving allied health professionals (e.g., physiotherapists, occupational therapists) and social professionals (e.g., psychologists, social workers) across care settings (e.g., primary care, rehabilitation centers) to gain a more comprehensive view of interprofessional collaboration. More specifically, future studies could include primary care to explore its crucial role in linking different aspects of integrated care. A third potential limitation of this study is the reliance on self-reported data. Respondents may have reported their intentions, perceptions, or preferences in ways that they believe are socially desirable or acceptable rather than what they genuinely do in practice. Future studies could address these limitations by incorporating behavioral data, such as direct observations or real-time logging of decisions and interactions, to complement self-reported measures and provide a more comprehensive understanding of professional practices in care complexity and collaboration.

## Implications and future directions

This study underlines the interdependency between care complexity and integrated care planning through interprofessional collaboration, within and between occupational groups and levels of work experience. While the selection of perspectives that need to be integrated during interprofessional collaboration is based on the interpretation of care complexity at hand, the recognition of care complexity also depends on expertise that allows professionals to pick up relevant information for optimal care delivery from different health and health-related domains [50,56]. This makes a shared understanding of care complexity encompassing all health domains critical to decide on the timing and composition interprofessional team within and across care settings. To optimize integrated care planning, tools or standard procedures could be designed and implemented to facilitate the establishment of a shared understanding of care complexity at hand which informs decision-making on the required interprofessional team and mode(s) of collaboration [57]. Moreover, (artificial intelligence-informed) models based on electronic health data and patient outcomes may function as decision-support tools that help to determine optimal timing and composition of MDTMs. The ICF model may provide a valuable framework for the tool development. For instance, a case-time series design following patients during hospitalization and discharge may shed light on key moments for MDTMs organized within and across settings to improve care transitions and reduce fragmentation in integrated care pathways. Finally, to be able to form a shared narrative and align expectations, educational interventions should be aimed at the development of a common vocabulary across occupations. Interprofessional education (IPE) and training could advocate for and use the same boundary object that affords the learning about, representation and transformation of knowledge to resolve problematic knowledge boundaries between professionals [49,58].

While person-centered care is gaining traction, the inclusion of family during MDTMs is not professionals’ standard preference or default procedure in clinical practice [57]. To better align family involvement in MDTMs with their preferences, short, standardized questionnaires could be developed to assess the desired level of participation in shared decision-making and communication. These questionnaires could help identify families who would benefit from direct participation in MDTMs, while for others, a patient advocate and tailored pre- and post-communication (e.g., recommendations, multiple choices, rationale) may be more appropriate. Additionally, to effectively involve families in MDTMs, educational interventions should focus on developing professionals’ competencies to adapt their language and interprofessional processes to meet family needs. Interprofessional education and training could incorporate simulated patients as part of the interprofessional team and include explicit evaluation of processes and outcomes from the patient’s perspective. This approach would not only enhance communication skills but also promote a more inclusive, family-centered care model.

To build on these findings, future research should investigate the processes that guide professionals’ decisions on care complexity, as well as subsequent care planning and delivery. While quantitative methods offer precision, the incorporation of qualitative interviews with participants could provide deeper insights into these decision-making processes and highlight potential differences across professions based on their unique roles and experiences. Therefore, future research employing a mixed-methods approach may further enrich the understanding of complex decision-making and improve the applicability to real-world settings. Additionally, cohort studies utilizing electronic health records and observational data could provide a clearer picture of actual behaviors and routines in clinical practice. This information would be instrumental in developing effective tools and standardized procedures related to the timing and composition, interaction and decision-making processes of MDTMs.

## Conclusion

This study deepens our multidimensional understanding of care complexity and integrated care planning by highlighting how professionals prioritize different health and health-related domains based on their expertise in a given setting. While some standard procedures seem to exist for including professionals within settings, cross-settings appear less structured. Notably, the inclusion of family members in MDTMs remains inconsistent. These findings can inform the development of decision-support tools and (inter-hospital or setting) policies to optimize the timing and composition of MDTMs in clinical practice. Furthermore, they can guide educational interventions to better prepare current and future professionals to deliver the right care, in the right place, at the right time. Future research may employ a mixed-method approach using interviews and observations to further enrich our understanding of complex decision-making processes in and across real-world settings.
